# TrkB Agonist Treatment Decreases Hippocampal Testosterone Contents in a Sex-Dependent Manner Following Neonatal Hypoxia and Ischemia

**DOI:** 10.3390/biom16020180

**Published:** 2026-01-23

**Authors:** Nur Aycan, Irem Isik, Nur Sena Cagatay, Feyza Cetin, Teresita J. Valdes-Arciniega, Burak Ozaydin, Sefer Yapici, Robinson W. Goy, Luc Collo, Qianqian Zhao, Jens Eickhoff, Peter Ferrazzano, Jon E. Levine, Amita Kapoor, Pelin Cengiz

**Affiliations:** 1 Department of Pediatrics, University of Wisconsin-Madison, Madison, WI 53792, USA; naycan@wisc.edu (N.A.); nursena.cagatay@gmail.com (N.S.C.); drfeyzacetin@gmail.com (F.C.); tere.valdes@wisc.edu (T.J.V.-A.); syapici@wisc.edu (S.Y.); ferrazzano@pediatrics.wisc.edu (P.F.); 2 Waisman Center, University of Wisconsin-Madison, Madison, WI 53705, USA; 3 University College Dublin, D04 V1W8 Dublin, Ireland; irem.ik@ucdconnect.ie; 4 Department of Neurological Surgery, University of Wisconsin-Madison, Madison, WI 53792, USA; burak-ozaydin@ouhsc.edu; 5 Wisconsin National Primate Research Center, University of Wisconsin-Madison, Madison, WI 53706, USA; rgoy@wisc.edu (R.W.G.); collo@wisc.edu (L.C.); jon.levine@primate.wisc.edu (J.E.L.); akapoor@wisc.edu (A.K.); 6 Department of Neuroscience, University of Wisconsin-Madison, Madison, WI 53706, USA; 7 Department of Biostatistics and Medical Informatics, University of Wisconsin-Madison, Madison, WI 53792, USA; qizhao@biostat.wisc.edu (Q.Z.); eickhoff@biostat.wisc.edu (J.E.)

**Keywords:** hypoxia ischemia, neonates, hippocampi, sex steroids, estradiol, testosterone, progesterone, corticosterone

## Abstract

Hypoxia–ischemia (HI)-related brain injury impacts millions of neonates worldwide. Male neonates are two times more susceptible to developing HI. We have previously reported that the administration of the neurotrophin receptor tyrosine kinase B (TrkB) agonist 7,8-dihydroxyflavone (DHF) following neonatal HI increases hippocampal TrkB phosphorylation and improves hippocampal-dependent learning and memory in early adult life only in females. We hypothesize that sex differences in HI outcomes are due to alterations in neonatal hippocampal steroid content, mainly the neural testosterone. At postnatal day 9, *C57BL/6J* mice underwent sham and Vannucci’s HI surgeries and were treated either with DHF or vehicle control. Hippocampi and plasma were collected on days 1 and 3 post-HI and liquid chromatography tandem mass spectrometry was used to determine the testosterone (T), estradiol (E_2_), progesterone (P_4_), and corticosterone (CORT) contents in these samples. All hippocampal steroid contents were at least 10-fold higher than in plasma, suggesting neural synthesis. Males had higher hippocampal T content than females at 3 days post-HI. Treatment with DHF reduced T in the female hippocampi at 3 days post-HI, but not in males. These findings suggest that the neuroprotective effect of DHF in females may be mediated, at least in part, through the reduction in hippocampal T following HI.

## 1. Introduction

Neonatal hypoxic–ischemic encephalopathy is an important health problem occurring in 1.5 cases per 1000 live births in developed nations [1,2]. Hypoxia–ischemia (HI)-related brain injury is characterized by a reduction in blood flow and consequently oxygen supply to the brain around the time of birth. Survivors of this condition can suffer several long-term neurological sequelae, including cerebral palsy, epilepsy, cognitive impairments, and behavioral disorders [3,4]. Epidemiological data including ~5500 hypoxic–ischemic newborns indicate that more than half of the cases occur in males, suggesting a possible sex-related vulnerability [5]. Similarly, preclinical studies have revealed a sex-specific susceptibility to HI, where male newborns are more susceptible to the long-term effects of HI [6,7,8].

We have previously reported that the administration of the neurotrophin receptor tyrosine kinase B (TrkB) agonist 7,8-dihydroxyflavone (DHF) after neonatal HI increases hippocampal TrkB phosphorylation and improves hippocampal-dependent learning and memory in early adult life in an estrogen receptor alpha (ERα)-dependent manner only in females [9,10,11]. Pre-treatment of cultured female hippocampal neurons with testosterone (T) inhibits the TrkB-mediated, ERα-dependent neuroprotective pathway in hippocampal neurons, reducing their survival under in vitro ischemic conditions [12]. While the mechanisms underlying these sex-specific vulnerabilities are unknown, recent studies have emphasized the role of local, brain-intrinsic mechanisms, such as the synthesis and action of neurosteroids, in mediating sex-specific responses to brain injury [13].

Testosterone plays a critical role in shaping brain development during the neonatal period, influencing neuronal differentiation, synaptic plasticity, and circuit organization [14]. In males, transient surges in circulating T occur within the first postnatal week, which can be locally converted to 17β-estradiol (E_2_) by aromatase within the brain, driving the masculinization of neural circuits [15]. Following HI, sex differences in hormonal regulation and receptor signaling have been reported to contribute to the differential vulnerability of male and female neonates [10,16]. Elevated T levels have been associated with increased susceptibility to oxidative stress and excitotoxic injury [17,18], whereas estrogenic signaling in females has been shown to confer neuroprotection through anti-inflammatory and trophic mechanisms [19,20]. In addition, T exposure increases the brain injury in a dose-dependent manner following global cerebral ischemia in a cardiac arrest model of mice [21]. Moreover, T exposure in female neonatal rats at birth worsens neurologic outcomes in female rodents after HI [22]. These observations suggest that alterations in endogenous T may critically influence sex-specific outcomes and responsiveness to neuroprotective interventions such as TrkB agonists post-HI.

It is worth noting that a body of experimental research indicates that sex steroids play an essential role in neuronal circuit formation and maintenance, via cross-talk with brain-derived neurotrophic factor (BDNF), a nerve growth factor [23]. In the hippocampus, BDNF as an intrinsic ligand to TrkB has a role in neuronal proliferation, differentiation, and homeostasis throughout the brain’s developmental stages. It has been established that estrogen regulates BDNF expression via both classical nuclear receptors and by the activation of membrane-associated ERs and subsequent upregulation of second messenger signaling systems [24]. It has been proposed that the estrogen/ER system is able to regulate the BDNF/TrkB system, while at the same time, the inhibitory effect of the TrkB/PI3K/AKT pathway on ERE transcription implies that the BDNF signal is able to limit and control BDNF expression [25]. While the progesterone receptor has been less studied, it has been shown that the activation of the classical nuclear progesterone receptor in neuronal cells increases BDNF synthesis [26].

In addition to estrogen, testosterone also has a indirect role in regulating the BDNF gene, via the activation of classical androgen receptors which upregulate androgen-responsive genes upstream of BDNF [27]. In addition, it is interesting that the BDNF/TrkB system positively regulates the expression of androgen receptors in neurons, enhancing testosterone’s regulation of the BDNF gene [28].

Besides TrkB, the TrkA receptor also has a role in the cross-talk between sex receptors and neurotrophins that may promote neuritogenesis. In unchallenged PC12 cells, both the synthetic androgen, R1881, and nerve growth factor (NGF) can promote neurite outgrowth via inducing the formation of the TrkA/androgen receptor/fliamin A complex and subsequent inactivation of RhoA. When the RhoA effector, ROCK, is pharmacologically inhibited, it promotes both androgen and NGF-induced neurite outgrowth, thus confirming the importance of TrkA in sex receptor/neurotrophin interaction [29].

It remains unknown whether the sex-specific neuroprotection conferred by DHF is mediated through brain-specific synthesis or regulation of T along with other sex steroids following HI. To address this gap, we examined circulating and hippocampal sex steroid contents in male and female neonatal mice at 1 and 3 days post-HI, with and without DHF treatment. Using an ultrasensitive liquid chromatography tandem mass spectrometry (LC-MS/MS) approach, we successfully quantified hippocampal T along with other sex steroid contents in the neonatal brain. Our findings reveal sex differences in hippocampal steroid profiles and demonstrate that DHF selectively reduces hippocampal T in females following HI, providing novel mechanistic insight into the hormonal underpinnings of female-specific neuroprotection mediated by TrkB activation.

## 2. Materials and Methods

### 2.1. Animal Use

All procedures were carried out in adherence with the NIH Guide for the Care and Use of Laboratory Animals using protocols reviewed and approved by the Institutional Animal Care and Use Committee at the School of Medicine and Public Health of the University of Wisconsin-Madison.

### 2.2. Induction of Neonatal HI

Mice were housed in ventilated cages, on a 12 h light/dark cycle with food and water available ad libitum. C57BL/6J mice with intact adrenal glands and gonads were used in the study since removal of the gonads and adrenals did not reduce steroids in any brain region in mice at birth [15]. Neonatal HI-related brain injury was induced at postpartum day (P) 9 using a modified version of a unilateral hypoxic–ischemic reperfusion injury model [30]. Briefly, mice were anesthetized with isoflurane (Butler Schein Animal Health Supply, Reno, NV, USA) (5% for induction, 3% for maintenance) in nitrous oxide/oxygen. The body temperature of the pups was maintained at 36 °C using a heated surgical table (Molecular Imaging Products, Bend, OR, USA). Under a surgical microscope (Nikon SMZ-800 Zoom Stereo, Nikon, Melville, NY, USA), a midline neck skin incision was made to visualize the left carotid sheath. The left common carotid artery was freed and electrocauterized with a bipolar electrocoagulator (Vetroson V-10 Bi-polar Electrosurgical Unit, Summit Hill Laboratories, Navesink, NJ, USA) and cut. The surgical site was flushed with 0.5% bupivacaine and closed with a single 6–0 silk suture. Mice were returned to their home cages with the dam. The cage was then placed into a normoxic chamber (36.5 °C) and monitored continuously for a 2 h recovery period. Following the 2 h recovery period, the mice pups were placed in a hypoxic chamber (BioSpherix Ltd., Redfield, NY, USA) equilibrated with 10% O_2_ and 90% N_2_ at 36.5 °C for 50 min. This is a well-characterized model of neonatal HI and results in hypoxic–ischemic reperfusion injury ipsilateral (IL) to the electrocauterized left common carotid artery while the contralateral side (CL) is only exposed to hypoxia only (HO) [11]. The sham-operated mice underwent the same procedure except electro-cauterization of the left common carotid artery or exposure to hypoxia [10].

### 2.3. Drug Administration

**Figure 1:**
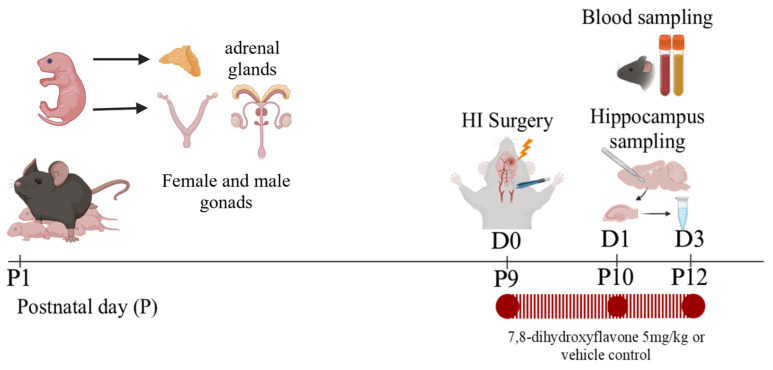
Experimental design.

Male and female littermates were randomly assigned to the sham-vehicle control or the HI +/− DHF therapy groups. DHF (Sigma, St. Louis, MO, USA) was dissolved in DMSO at a concentration of 3 mg/mL and put into aliquots that could be frozen for up to a month. On the day of administration, the DHF was diluted to 0.1 mg/mL with sterile 0.01 M PBS and injected intraperitoneally at a final concentration of 5 mg/kg. The HI + DHF-treated mice received the initial dose of the drug at 10 min post-HI followed by daily doses until sacrifice. The HI-vehicle control and sham-vehicle control groupsreceived an equal volume of 0.01 M PBS for the same duration (Figure 1).

### 2.4. Blood Sampling and Hippocampal Tissue Extraction

Approximately 200–300 µL of blood was drawn directly from P10 and P12 mice via sterile cardiac puncture under anesthesia. The blood was immediately transferred into blood tubes containing EDTA to prevent clotting and placed on wet ice. The samples were then centrifuged at 2200× *g* for 3 min at 4 °C to separate the plasma from the cellular components. The plasma was carefully transferred to fresh microtubes and stored at −80 °C for subsequent analysis [31].

Following blood sampling, the hippocampi were extracted as described previously by our laboratory [32]. Briefly, following decapitation, the whole brain was extracted and placed in a Petri dish in a dorsal orientation. Using curved forceps, the cerebellum and brainstem were removed. Then a sagittal incision was made to separate the hemispheres [33]. The basal ganglia and thalamus were then removed from the medial surface of the hemisphere [34,35,36]. Both hippocampi were then identified and harvested [37,38]. The hippocampi (IL and CL) from two mice (n = 1) were separately pooled and stored at −80 °C to optimize the LC-MS/MS measurements (Figure 1).

### 2.5. LCMS-MS Measurement

The use of LC-MS/MS offers several advantages over the traditional radioimmunoassay methods used in previous studies measuring brain-based steroids in neonatal rodent brains [15]. LC-MS/MS provides increased sensitivity, allowing for the measurement of low concentration steroids; this becomes especially vital in the context of assessing brain-based steroid content [39]. Moreover, LC-MS/MS allows for the quantification of multiple hormones from a single sample with a superior specificity in steroid detection, eliminating the nonspecific antibody interactions of other methods [15,40]. All the measurements were performed on coded samples blinded to the experimenter.

Plasma samples for testosteron (T), estradiol (E_2),_ progesterone (P_4)_, corticosterone (CORT), androstenedione, and estrone were measured using a previously published LC-MS/MS method [41,42]. The detailed method to extract and measure T, E_2,_ P_4_, CORT, androstenedione, and estrone in hippocampal tissue is contained in the Supplementary Materials. Briefly, hippocampal tissue was homogenized and then purified. Steroid extraction and sample cleanup on the supernatant were achieved using Oasis HLB solid phase extraction (Waters Corporation, Milford, MA, USA). The samples were then analyzed using a 6500+ triple quadrupole (Sciex, Toronto, ON, Canada) adapted from the serum method. Intra- and inter-assay CV was assessed with a pool of mouse hippocampi and ranged from 1.7 to 8.7 and 8.9 to 19.3%, respectively. Limits of detection (LODs) for T, E_2_, P_4_, and CORT for the hippocampi were 0.08, 0.004, 0.0036, and 0.7 pg/mg, respectively. For the plasma samples, the LODs for T, E_2_, P_4_, and CORT were 0.003, 0.001, 0.003, and 0.2 pg/mL, respectively.

### 2.6. Statistical Analysis

In samples where a hormone was not detected, the LOD value was assigned. This could artificially increase mean values but was taken into consideration during the analysis. Extreme values were inspected using univariate analysis with histogram to check for data errors and removal of outliers. One measurement from T and another from CORT were excluded due to being outliers. Additionally, we do not report the contents for androstenedione and estrone because they were undetectable in all samples. The data was log transformed, but did not pass normality or non-equal variance testing. Thus, we used analysis of variance (ANOVA) because it is robust to moderate violations of normality and non-equal variance. Multi-factorial ANOVA was conducted to evaluate hormone differences between treatment groups, age, and sex. Specific sliced interaction effect contrasts were constructed to conduct an analysis of the simple effects for age, sex, and treatment. Similarly, for hippocampi, the impact of treatment, age, sex, and side in sham, HI, and HI + DHF mice was analyzed using four-way ANOVA, and sliced interaction effect contrasts were constructed to conduct an analysis of the simple effects for age, sex and side in sham, HI, and HI + DHF mice using four-way ANOVA. The results were summarized in terms of means and standard errors (SEMs) (Table 1). The associations between all reported *p*-values are two-sided, and *p* < 0.05 was used to define statistical significance (Table 1). Although the sample sizes may be too small to detect very subtle differences, we believe that the four-way ANOVA, along with the corresponding sliced interaction contrasts, provides the most sensitive approach for the comparisons of interest. This modeling strategy offers a comprehensive framework and results in smaller standard errors. Statistical analyses were conducted using SAS software (SAS Institute, Cary, NC, USA), version 9.4.

## 3. Results

The ANOVA *p*-values for experimental group and age comparisons within each sex are displayed in the figures with * denoting a significant difference between the corresponding sex. 

### 3.1. Multivariate Analysis for T

Multivariate analysis of plasma T content revealed a significant effect of treatment F_(2, 108)_ = 4.69, *p* = 0.01), sex (F_(1, 108)_ = 35.7, *p* < 0.001), age (F_(1, 108)_ = 9.62, *p* = 0.003), and agebysex interaction (F_(2, 108)_ = 7.04, *p* = 0.001). In the hippocampus, there was a significant effect of sex (F_(1, 146)_ = 14.9, *p* = 0.002), age (F_(1, 146)_ = 65.9, *p* = < 0.001), and age by sex interaction (F_(1, 146)_ = 5.46, *p* = 0.021).

### 3.2. T Contents in Plasma and Hippocampi in Sham Mice

**Figure 2:**
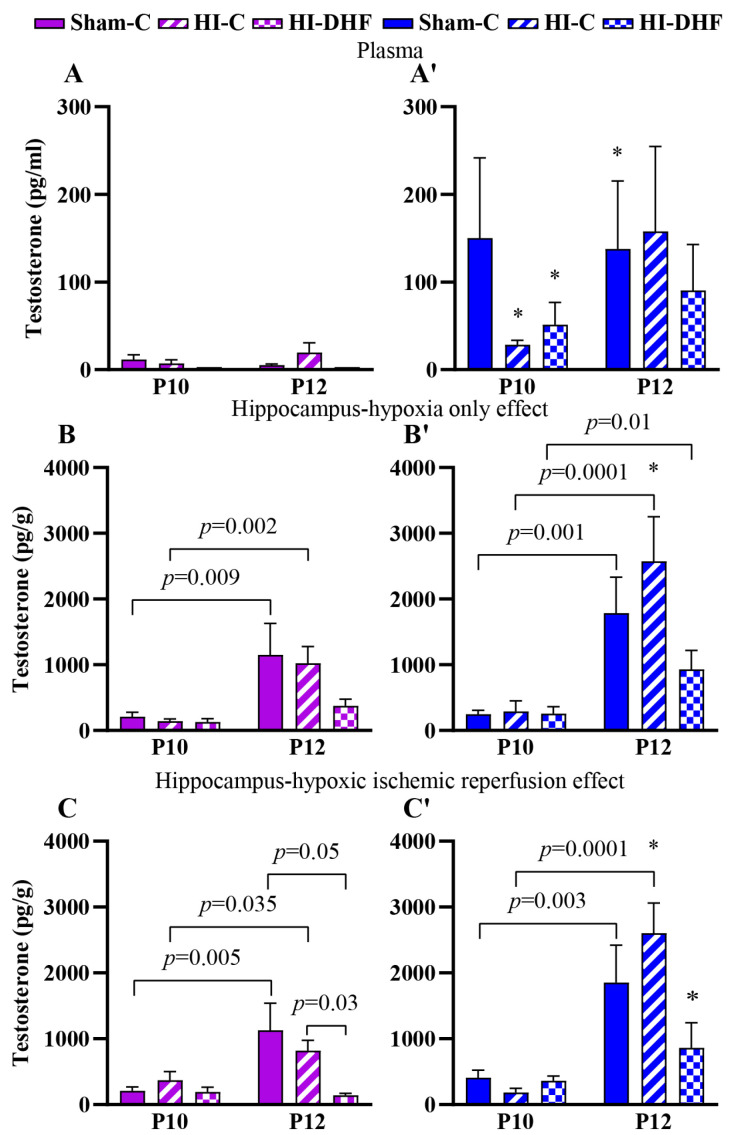
T contents in plasma and hippocampi at P10 and P12 post-HI in both sexes. Pink—female; blue—male. Plasma T, (**A**,**A′**). HO hippocampal T, (**B**,**B′**). HI hippocampal T, (**C**,**C′**). Data are presented as mean ± SEM. Statistical analysis was performed using four-way ANOVA. *p*  <  0.05 was considered statistically significant. * indicates a significant difference compared with the corresponding female treatment group (n = 3–14).

Circulating T contents remained relatively stable between P10 and P12, with no differences observed across experimental groups (Figure 2A,2A′). Hippocampal T contents were approximately 10-fold higher than circulating contents, which suggests the presence of local steroid synthesis, neurostereidogenesis, or regulatory mechanisms (Figure 2A,A′ vs. Figure 2B,B′,C,C′). Hippocampal T content was higher in P12 compared to P10 sham mice, regardless of laterality in both males and females (Figure 2B,B′,C,C′).

### 3.3. Effect of HO and DHF on Hippocampal T Contents

In hippocampi that were exposed only to hypoxia, the T contents at a given time point in either sex was not statistically significantly different between experimental groups. However, T contents in P12 CL hippocampi were higher than P10 regardless of sex in every experimental group. There was a sex-specific difference in CL hippocampal T content at P12, where male hippocampi show higher T content compared to females (*p* = 0.02) (Figure 2B,B′).

### 3.4. Effect of HI and DHF on Hippocampal T Contents

In hippocampi that were exposed to HI (IL), the T contents at the P10 time point in either sex was not different between experimental groups. Similarly to the CL side, there was an increase in T in sham and IL hippocampi at the P12 time point in both sexes. However, the increase in male IL hippocampi was larger than in female IL hippocampi (*p* = 0.01). Interestingly, with DHF treatment, there was a significant decrease in the T content in the female hippocampi at P12 but not in male hippocampi (*p* = 0.03). These findings suggest that DHF treatment may attenuate HI-induced hippocampal T elevation in females (Figure 2C,C′).

### 3.5. Multivariate Analysis for E_2_

Multivariate analysis of plasma E_2_ content revealed a significant effect of treatment F_(2, 108)_ = 172, *p* < 0.001), sex (F_(1, 108)_ = 12.7, *p* < 0.005), age (F_(1, 108)_ = 55.3, *p* < 0.0001), treatment by sex interaction (F_(2, 108)_ = 5.97, *p* = 0.004), treatment by age (F_(2, 108)_ = 132, *p* < 0.0001), sex by age interaction (F_(1, 108)_ = 9.4, *p* = 0.003), and treatment by age by sex interaction (F_(2, 108)_ = 3.97, *p* = 0.02). In the hippocampus, there was a significant effect of treatment (F_(2, 147)_ = 6.63, *p* = 0.0017), treatment x age (F_(1, 147)_ = 6.1, *p* = 0.003), and treatment by sex interaction (F_(1, 147)_ = 4.7, *p* = 0.033).

### 3.6. E_2_ Contents in Plasma and Hippocampi in Sham Mice

**Figure 3:**
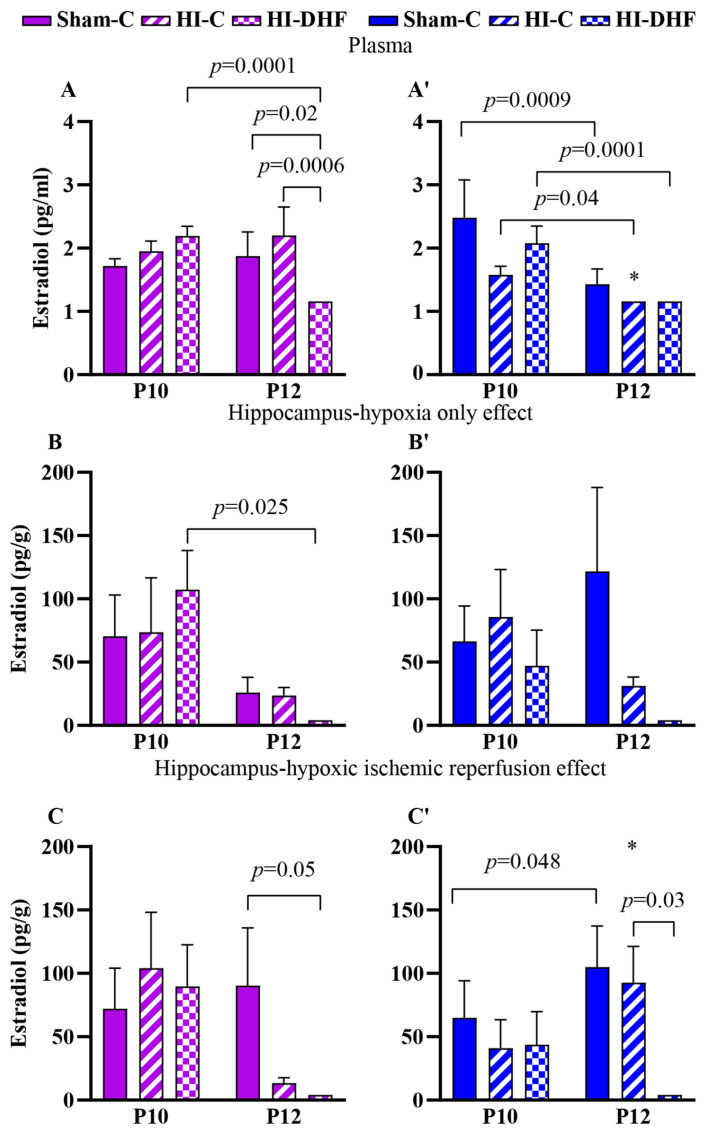
E_2_ contents in plasma and hippocampi at P10 and P12 post-HI in both sexes. Pink—female; blue—male. Plasma T, (**A**,**A′**). HO hippocampal E_2_, (**B**,**B′**). HI hippocampal E_2_, (**C**,**C′**). Data are presented as mean ± SEM. Statistical analysis was performed using four-way ANOVA. *p*  <  0.05 was considered statistically significant. * indicates a significant difference compared with the corresponding female (n = 3–14).

Hippocampal E_2_ content was approximately 100-fold higher than circulating contents, suggesting that E_2_ contents in the hippocampi are dependent on intrinsic neurosteroidogenesis or region-specific regulatory mechanisms (Figure 3A,A′ vs. Figure 3B,B′,C,C′). Developmental changes in circulating E_2_ were observed in sham males, with a significant reduction from P10 to P12 that was not observed in female sham hippocampi (Figure 3A,A′). In contrast, hippocampal E_2_ content increased from P10 to P12 in sham males (Figure 3C,C′). No significant developmental changes were found in either plasma or hippocampal E_2_ content in sham females (Figure 3A–C).

### 3.7. Effect of HO and DHF on Hippocampal E_2_ Content

In CL hippocampi that are exposed only to hypoxia, there was a minimal impact on hippocampal E_2_ content at P12. A significant reduction in hippocampal E_2_ was seen at P12 compared to P10 in the HI + DHF female group (Figure 3B,B′).

### 3.8. Effect of HI and DHF on Hippocampal E_2_ Content

Following hypoxia–ischemia–reperfusion injury, there was a more pronounced difference in the hippocampi. At P12, hippocampal E_2_ contents were significantly higher in sham females (*p* = 0.05) and males (*p* = 0.01) compared to their HI + DHF counterparts. IL males also had higher hippocampal E_2_ contents than HI + DHF males, indicating the suppression of E_2_ following HI and DHF treatment, particularly in males. Interestingly, at P12, IL male hippocampi displayed increased E_2_ content compared to their female counterparts, highlighting a clear sex difference (*p* = 0.02) (Figure 3C,C′).

### 3.9. Multivariate Analysis for P_4_

Multivariate analysis of plasma P_4_ content revealed a significant effect of treatment x age (F_(1, 108)_ = 20.1, *p* < 0.0001), treatment x sex (F_(2, 108)_ = 12.5, *p* < 0.0001), and sex x age (F_(2, 108)_ = 8.96, *p* = 0.003). In the hippocampus, there was a significant effect of age (F_(1, 147)_ = 57.6, *p* < 0.0001) and age x sex (F_(1, 147)_ = 6.63, *p* = 0.011).

### 3.10. P_4_ Content in Plasma and Hippocampi in Sham Mice

**Figure 4:**
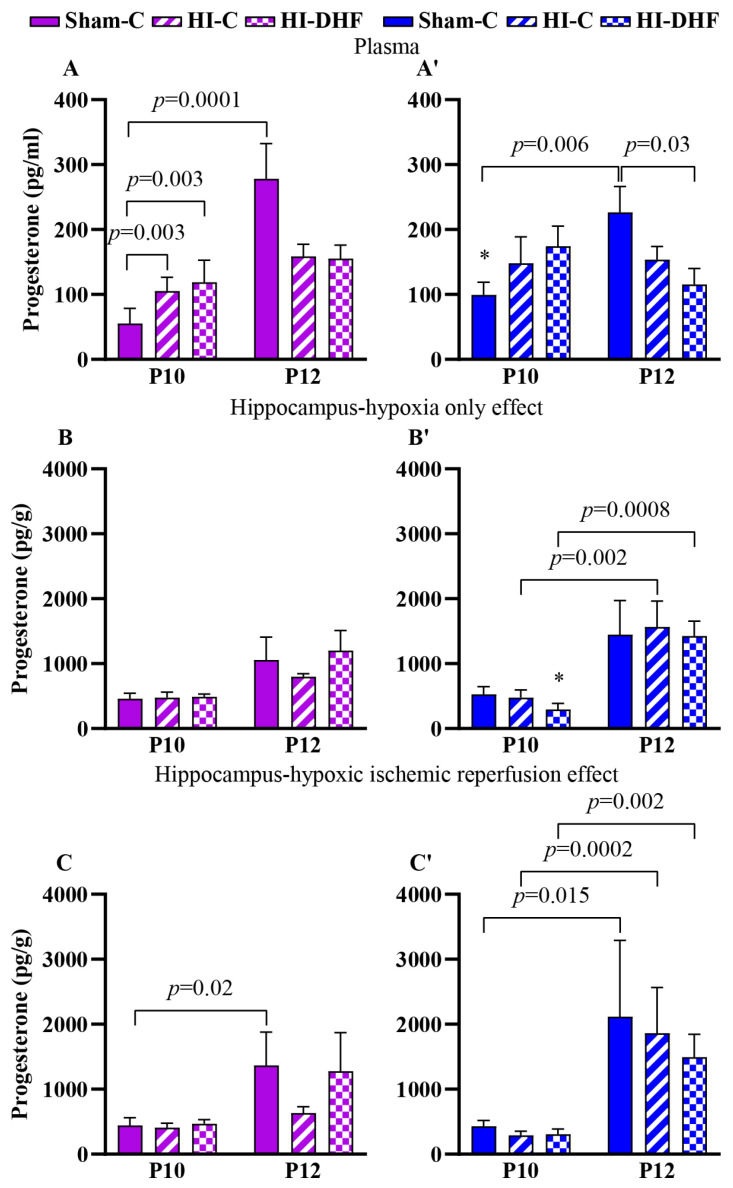
P_4_ content in plasma and hippocampi at P10 and P12 post-HI in both sexes. Pink—female; blue—male. Plasma P_4_, (**A**,**A′**). HO hippocampal P_4_, (**B**,**B′**). HI hippocampal P_4_, (**C**,**C′**). Data are presented as mean ± SEM. Statistical analysis was performed using four-way ANOVA. *p*  <  0.05 was considered statistically significant. * indicates a significant difference compared with the corresponding female (n = 3–14).

Circulating P_4_ significantly increased from P10 to P12 in sham females and males, with higher plasma contents in sham males than females at P10 (*p* = 0.003; Figure 4A,A′). Hippocampal P_4_ in sham mice also increased from P10 to P12 in both sexes (Figure 4C,C′).

### 3.11. Effect of HO and DHF on Hippocampal P_4_ Content

No changes in hippocampal P_4_ were observed at either the P10 or P12 time points under HO with or without DHF, suggesting relative stability of local P_4_ despite injury or treatment. However, at P10, P_4_ contents were significantly higher in females than males (*p* = 0.025) in hypoxic only hippocampi, suggesting early sex-specific elevation of P_4_ with DHF. P_4_ contents significantly increased from P10 to P12 in the hippocampi of HO male mice, both with and without DHF treatment (Figure 4B,B′).

### 3.12. Effect of HI and DHF on Hippocampal P_4_ Content

A similar pattern to the hypoxic only hippocampi was seen in the IL hippocampi. There was a significant increase in P_4_ in HI and HI + DHF hippocampi between P10 and P12 males (Figure 4C,C′), indicating robust developmental upregulation across groups.

### 3.13. Multivariate Analysis for CORT

Multivariate analysis of plasma CORT content revealed a significant effect of age x sex (F_(2, 108)_ = 6.66, *p* = 0.0019). In the hippocampus, there was a significant effect of treatment (F_(2, 147)_ =26.2, *p* < 0.0001), age (F_(1, 147)_ = 114, *p* < 0.0001), treatment x age (F_(1, 147)_ = 31.3, *p* < 0.0001), age x sex (F_(2, 147)_ = 4.0, *p* = 0.05), and treatment x age x sex (F_(2, 147)_ = 3.72, *p* = 0.03).

### 3.14. CORT Contents in Plasma and Hippocampi in Sham Mice

**Figure 5:**
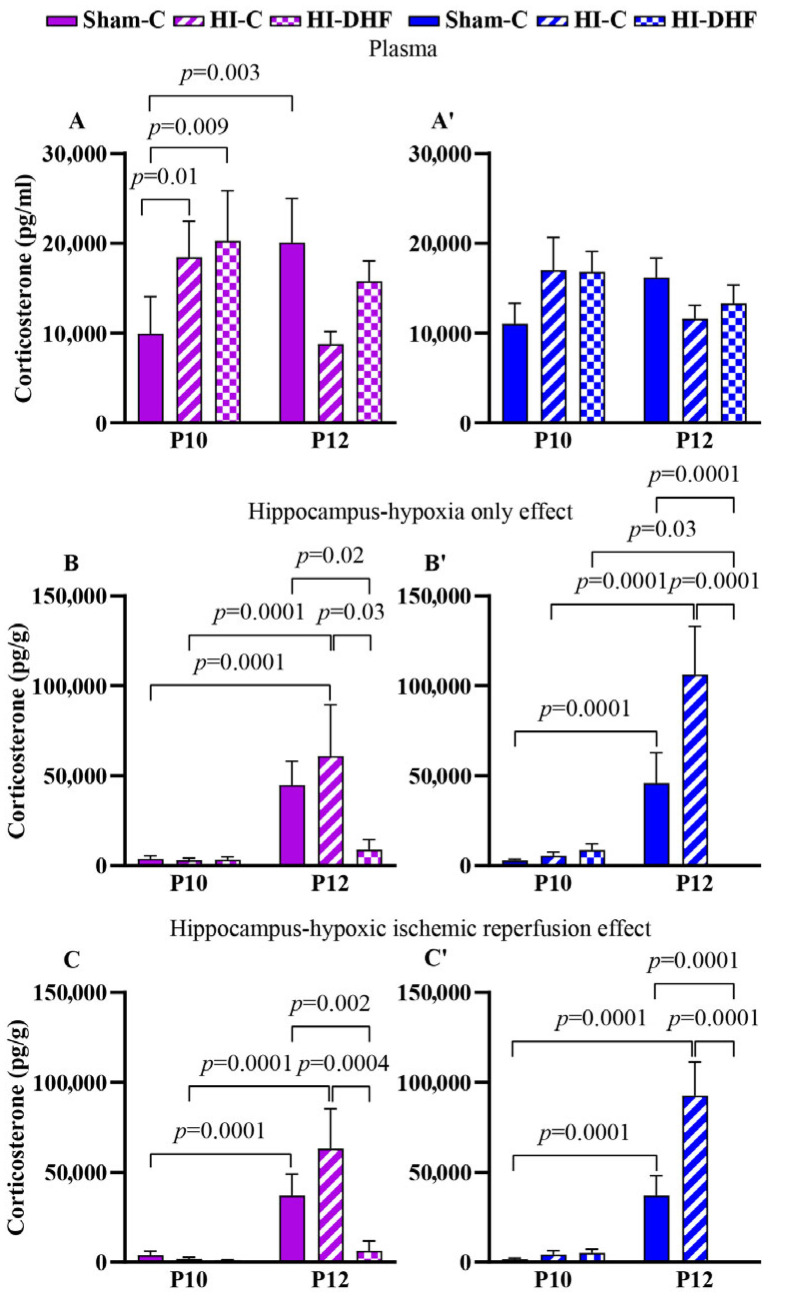
CORT content in plasma and hippocampi at P10 and P12 following hypoxic–ischemic injury in both sexes. Pink—female; blue—male. Plasma CORT, (**A**,**A′**). HO hippocampal CORT, (**B**,**B′**). HI hippocampal CORT, (**C**,**C′**). Data are presented as mean ± SEM. Statistical analysis was performed using four-way ANOVA. *p*  <  0.05 was considered statistically significant (n = 3–14).

In plasma, CORT content increased significantly between P10 and P12 in sham females but not in males (Figure 5A,A′). In the hippocampi, CORT content increased from P10 to P12 in sham females and males at P12 compared to P10 (Figure 5B,B′,C,C′). Further research is needed to understand the source to these changes in sham hippocampi.

### 3.15. Effect of HO and DHF on Hippocampal CORT Contents

There was a significant increase in CL hippocampal CORT content in both females and males between the P10 and P12 time points. Interestingly, at the P12 time point, there was a remarkable decrease in the CORT content in response to DHF treatment in both male and female CL hippocampi. This suggests a marked suppression of hippocampal CORT under DHF conditions (Figure 5B,B′).

### 3.16. Effect of HI and DHF on Hippocampal CORT Contents

As with the CL hippocampi, increases in IL hippocampi were noted in both sexes, indicating a consistent age-related hippocampal accumulation of CORT regardless of sex. In addition, there was a large and significant decrease in CORT content in IL hippocampi in response to DHF treatment (Figure 5C,C′). These results suggest that DHF treatment post-HI suppresses hippocampal CORT content in both female and male mice. Despite these injury-related effects, no consistent sex differences were detected in hippocampal or circulating CORT content at either time point or condition.

## 4. Discussion

Sex steroid hormones, including androgens (T, dihydrotestosterone, and androstenedione), estrogens (E_2_, estrone, and estriol), and P_4_, exert their effects through nuclear and membrane-associated receptors, modulating both genomic and rapid non-genomic signaling pathways. Traditionally attributed to circulating sex steroids, it has been shown that brain differentiation involves locally synthesized neurosteroids, such as T and E_2_. These brain-derived steroids influence neurogenesis, synaptic plasticity, and behavior, enabling the precise regulation of brain development and function [13]. In fact, the perinatal hormone environment programs the development of some neuronal circuitries that support sex-specific behaviors and physiological functions in adulthood [11,15,43].

One crucial difference between sexes is that while female mice have low T contents throughout the embryonic and perinatal periods, male mice experience two significant gonadal T surges. In mice, the first surge occurs during embryonic day 13, driving gonadal development. The second one, the perinatal surge (0–4 h postnatally) influences the masculinization of neuronal morphology and behavior [44]. T is converted into E_2_ by the enzyme aromatase, which is expressed in various brain regions including the hippocampus, hypothalamus, and cortex [13,45]. In neonatal rats, the E_2_ produced by neurons plays a crucial role in permanently masculinizing the hypothalamus and preoptic area, organizing the neural networks that control male sexual behavior and gonadotropin secretion throughout adulthood. The sex steroid content varies across different brain regions during postnatal development, indicating that local steroidogenesis within the brain contributes to the process of sexual differentiation [15].

The present study investigated the effect of the TrkB agonist DHF on the plasma and hippocampal sex steroid contents in neonatal male and female mice utilizing LC-MS/MS following neonatal HI-related hippocampal injury. Our data demonstrate that DHF decreases hippocampal T content selectively in females compared to males at an early time point (3 days post-HI). This sex-specific effect suggests that TrkB activation may differentially interact with local steroidogenic pathways in the injured neonatal brain. In addition, the observation that hippocampal sex steroid contents were markedly higher than plasma contents supports the concept that neonatal brain injury can stimulate endogenous neurosteroid production or metabolism within the hippocampus. Together, these findings emphasize the importance of de novo neurosteroid synthesis as a key component of the neuroendocrine response to hypoxia–ischemia and point to T regulation as a potential mechanism underlying the female-specific neuroprotection observed with DHF treatment.

In our study, we found that there was no change in the plasma T content between P10 and P12 in either sex. However, there was a significant decrease in plasma contents of E_2_ between P10 and P12 in male mice. In contrast, there was a significant increase in plasma P_4_ between P10 and P12 in both male and female mice. The CORT plasma contents also increased significantly between P10 and P12 only in female sham mice but not male sham mice. The reasons for the changes in circulating sex hormone contents in sham mice between P10 and P12 are unknown. Possible reasons include normal developmental changes in mice, or the changes could be secondary to anesthesia-induced neuroinflammation subsequent to surgery at P9 [46]. Further work that includes additional developmental points and naïve mice are needed to address this question.

High brain content of sex steroid hormones are mainly found in regions significantly affected by neonatal HI, such as the hippocampus, a key structure involved in memory, behavior, and neurodevelopmental regulation [45]. The hippocampus is highly sensitive to both acute and chronic hypoxic insults; damage to this region has been consistently associated with long-term behavioral and cognitive impairments in both experimental [47,48,49,50,51,52,53] and human studies [54,55,56]. While the role of sex in cellular responses remains unclear, sex-specific brain steroid profiles offer insights into the interplay between neurodevelopment and sexual differentiation. Sex-specific profiles of neurosteroids in the hippocampus underscore its unique role in neurodevelopment and sexual differentiation, justifying the direct measurement of local hormone contents.

Here we report both the effect of hypoxia alone and HI on hippocampal neurosteroid contents between P10 and P12 with and without DHF treatment. There was no difference between CL and IL hippocampi contents within any of neurosteroids at either P10 or P12 regardless of DHF treatment for both sham and injured mice. The one exception is that DHF treatment resulted in a decrease in T contents in female IL hippocampi that was absent in female CL hippocampi at P12. Numerous reports indicated that increased T exacerbates post-ischemia neuropathology [8]. Thus, this result points to a possible sex-specific neuroprotective effect of TrkB activation in females.

It has been reported that DHF decreases serum CORT in a dose-dependent manner in rats subjected to chronic mild stress [57]. While we saw no effect of DHF on plasma contents, it is interesting that DHF dramatically decreased CORT hippocampal contents in both HO and HI hippocampi at the P12 time point regardless of sex. Adult mice subjected to 2 h of immobilization exhibited increased CORT and stress-induced reduction in spatial learning that was rescued by the potentiation of the TrkB pathways via DHF [58]. In adolescent mice (P31), DHF blocks the CORT-induced errors in reverse learning tasks in females and improves the performance of male mice [59]. CORT exposure during adolescence causes a shift in the balance between the full-length TrkB and a truncated, inactive form, favoring the truncated form throughout multiple corticolimbic brain regions including the medial prefrontal cortex (mPFC), ventral hippocampus (vHC), amygdala, and ventral striatum. This shift reduces the ratio of full-length to truncated TrkB, thereby decreasing the ability of full-length TrkB to initiate intracellular signaling [60]. Thus, the DHF-induced decrease in hippocampal CORT we observed post-HI may preserve TrkB neuroprotective signaling.

It has been reported that DHF has a moderate inhibitory effect on aromatase, the enzyme that catalyzes the conversion of T to E_2_ [61,62]. We observed that in female mice, DHF treatment post-HI resulted in a decrease in E_2_ at P12 compared to P 10 in IL hippocampi. DHF treatment also resulted in a decrease in male IL hippocampi E_2_ at P12 compared to vehicle control treated male mice post-HI. While the loss of neuron-derived estrogen in aromatase knock-out mice causes significant impairment in hippocampal-dependent cognitive functions [63], it is unclear whether the reduction in hippocampal estrogen in neonatal mice we report is sufficient to cause significant neuropathology in our model. Further work is required to better understand the effect of DHF on aromatase activity and E_2_ contents post-neonatal HI.

## 5. Limitations and Future Directions

There are several limitations to this study that need to be addressed. First, we cannot rule out that the observed effects of DHF are not only TrkB-dependent but an off-target consequence of the drug. In future experiments, we plan to use the TrkB antagonist ANA-12 post-HI to address this issue. Second, in this study, we do not directly measure aromatase expression. Future studies will include the investigation of hippocampal aromatase levels or activity to improve the mechanistic strength of our conclusions. Third, the link between reduced T and neuroprotection in females could be confirmed by applying exogenous T in the presence of DHF post-HI to see if neuroprotection is lost. Future directions in our research will also include experiments to examine the effect of TrkB activation on cytoskeletal pathways such as RhOA, which could influence injury responses and explain the sexually differential effect of DHF [29]. In addition, investigating the effect of DHF on possible cross-talk between androgen receptors and cytoskeletal scaffolding proteins post-HI could illuminate the interaction between Trk receptors (TrkB and TrkA) and sex steroids [23,29]. It has also been reported that, in PC12 cells, TrkA regulates neurite outgrowth through interaction with a complex that includes the androgen receptor and the signaling effector filamin A.. The complex has a role in regulating GTPase signaling and inducing neuritogeneis in these cells [64]. In addition, this study cannot differentiate between cell types in the hippocampus. Further experiments using single nuclear RNA sequencing would be invaluable in determining the relative contributions of neurons and glial cells to our observations.

## 6. Conclusions

To our knowledge, this is the first study to use the ultrasensitive LC-MS/MS method to study hippocampal neurosteroid contents in neonatal mice post-HI. We have shown that at day 3 following neonatal HI, there was female-specific reduction in T in response to DHF in the hypoxic–ischemic hippocampi. Thus, there may be a role for TrkB signaling in altering neurosteroid profiles in the post-HI hippocampi resulting in female-biased neuroprotection. DHF also modulated CORT contents at 3 days post-HI in neonatal mice in our study. Given the inhibitory effect of CORT on TrkB signaling, this may be an important aspect of DHF-mediated neuroprotection.

While the role of sex in cellular responses remains unclear, the sex-specific brain steroid profiles we report here may offer insights into the interplay between neurodevelopment and sexual differentiation. Sex-specific profiles of neurosteroids in the hippocampus underscore its unique role in neurodevelopment and sexual differentiation, justifying further studies involving the direct measurement of local hormone contents.

## Figures and Tables

**Table 1:** The plasma and hippocampal contents of each hormone across sexes and experimental groups.

			Sham-C			HI-C			HI-DHF		
			P10	P12	*p*-Value	P10	P12	*p*-Value	P10	P12	*p*-Value
Testosterone	Female	Plasma	11.6 ± 5.3 (8)	5.3 ± 1.2 * (9)	0.64	7.1 ± 4.2 * (8)	19.6 ± 11.1 (9)	0.27	2.9 ± 0.0 * (8)	2.9 ± 0.0 (14)	1.00
		CL	211 ± 67.9 (8)	1152 ± 476 (9)	0.01	140 ± 36.9 (5)	1023 ± 253 * (10)	0.002	130.0 ± 48.6 (8)	376 ± 102 (3)	0.057
		IL	210 ± 58.4 (8)	1129 ± 414 (8)	0.005	370. ± 133 (6)	821 ± 157 * (9)	0.04	195 ± 68.9 (8)	142 ± 30.5 * (3)	0.94
	Male	Plasma	150 ± 91.3 (8)	137.8 ± 77.6 * (12)	0.59	28.5 ± 5.1 * (8)	157.7 ± 97 (14)	0.75	51.5 ± 25.3 * (8)	90.4 ± 52.6 (14)	0.08
		CL	249 ± 58.2 (8)	1788 ± 545 (8)	0.001	292 ± 1601 (8)	2574 ± 678 * (7)	<0.0001	255 ± 107 (8)	931 ± 287 (3)	0.01
		IL	409 ± 114.7 (8)	1853 ± 570.1 (9)	0.003	186 ± 60.8 (8)	2601 ± 460 * (7)	<0.0001	363 ± 72.9 (8)	861 ± 380 * (3)	0.16
Estradiol	Female	Plasma	1.7 ± 0.1 (8)	1.9 ± 0.4 (9)	0.8821	2.0 ± 0.2 (8)	2.2 ± 0.5 * (9)	1.00	2.2 ± 0.2 (8)	1.2 ± 0.0 (14)	<0.0001
		CL	70.5 ± 32.5 (8)	26.0 ± 12.1 (9)	0.80	73.6 ± 43.0 (5)	23.7 ± 6.4 (10)	0.89	107 ± 30.9 (8)	4.1 ± 0.0 (3)	0.03
		IL	72.1 ± 31.9 (8)	90.3 ± 45.7 (8)	0.28	104.2 ± 44.1 (6)	13.5 ± 4.2 * (9)	0.15	89.8 ± 32.8 (8)	4.1 ± 0.0 (3)	0.07
	Male	Plasma	2.5 ± 0.6 (8)	1.4 ± 0.2 (12)	0.0009	1.6 ± 0.1 (8)	1.2 ± 0.0 * (14)	0.04	2.1 ± 0.3 (8)	1.2 ± 0.0 (14)	0.0001
		CL	66.4 ± 28.1 (8)	122 ± 66.5 (9)	0.36	85.7 ± 37.5 (8)	31.3 ± 7.1 (7)	0.88	47.1 ± 28.2 (8)	4.1 ± 0.0 (3)	0.36
		IL	64.9 ± 29.3 (8)	105 ± 32.4 (9)	0.05	41.0 ± 22.5 (8)	92.7 ± 28.5 * (7)	0.06	43.8 ± 26.0 (8)	4.1 ± 0.0 (3)	0.37
Progesterone	Female	Plasma	55.4 ± 23.2 * (8)	278 ± 54 (9)	<0.0001	105 ± 21.6 (8)	158 ± 18.7 (9)	0.14	119 ± 33 (8)	155 ± 20.8 (14)	0.19
		CL	460 ± 85.2 (8)	1057 ± 351.4 (9)	0.10	477 ± 82.8 (5)	799 ± 48.5 (10)	0.26	488 ± 44.1 * (8)	1203 ± 307 (3)	0.16
		IL	445 ± 117.2 (8)	1368 ± 512.2 (8)	0.02	410 ± 67.8 (6)	633 ± 96.9 (9)	0.39	467 ± 62.9 (8)	1278 ± 594 (3)	0.15
	Male	Plasma	99.4 ± 19.5 * (8)	227 ± 39.5 (12)	0.007	148 ± 40.6 (8)	153 ± 20.3 (14)	0.59	175 ± 30.8 (8)	115 ± 24.7 (14)	0.13
		CL	526 ± 119 (8)	1448 ± 525 (9)	0.096	476 ± 118 (8)	1567 ± 398.8 (7)	0.002	294 ± 92.0 * (8)	1426 ± 231 (3)	0.0008
		IL	431 ± 88.1 (8)	2115 ± 1174 (9)	0.015	290 ± 63.8 (8)	1863 ± 703.9 (7)	0.0002	307 ± 81.1 (8)	1496 ± 350 (3)	0.002
Corticosterone	Female	Plasma	9917 ± 4145 (8)	20,068 ± 4915 (9)	0.003	18,446 ± 4001 (8)	8788 ± 1397 (9)	0.08	20,244 ± 5602 (8)	15,778 ± 2244 (14)	0.51
		CL	3811 ± 1673 (8)	44,778 ± 13,265(9)	<0.0001	2891 ± 1364 (5)	60,889 ± 28,627 (9)	<0.0001	3331 ± 1572 (8)	8768 ± 5829 (3)	0.23
		IL	3939 ± 2114 (8)	37,027 ± 11,984(8)	<0.0001	1780 ± 1066 (6)	63,159 ± 22,218 (9)	<0.0001	1040 ± 326 (8)	6248 ± 5534 (3)	0.27
	Male	Plasma	11,040 ± 2289(8)	16,207 ± 2161(12)	0.16	17,005 ± 3651(8)	11,603 ± 1509 (14)	0.60	16,819 ± 2253 (8)	13,294 ± 2058 (14)	0.31
		CL	2808 ± 836 (8)	45,767 ± 17,085 (9)	<0.0001	5509 ± 2020 (8)	106,172 ± 26,835 (7)	<0.0001	8699 ± 3528 (8)	714 ± 0.0 (3)	0.03
		IL	1650 ± 614 (8)	37,119 ± 10,967 (9)	<0.0001	4248 ± 2252 (8)	92,522 ± 18,835 (7)	<0.0001	5187 ± 2118 (8)	714 ± 0.0 (3)	0.12

Table 1 presents the mean ± SEM contents for the sex steroid hormone analytes, with * denoting a significant difference between the corresponding sex.

## Data Availability

All reasonable requests for access to our data will be honored.

## Supplementary Materials

The following supporting information can be downloaded at: https://www.mdpi.com/article/10.3390/biom16020180/s1, Supplemental Method of LCMS-MS Measurement.

## Abbreviations

The following abbreviations are used in this manuscript:

HI	Hypoxia–ischemia
HO	Hypoxia only
P	Postnatal day
TrkB	Tyrosine kinase B receptor
DHF	7,8 dihydroxyflavone
LC-MS/MS	Liquid chromatography-tandem mass spectrometry
ERα	Estrogen receptor alpha
T	Testosterone
E_2_	Estradiol
P_4_	Progesterone
CORT	Corticosterone
CL	Contralateral
IL	Ipsilateral
