# Supplementary material for: TrkB Agonist Treatment Decreases Hippocampal Testosterone Contents in a Sex-Dependent Manner Following Neonatal Hypoxia and Ischemia
Source: Biomolecules. 2026 Jan 23;16(2):180. doi: 10.3390/biom16020180 (PMC12938734; doi:10.3390/biom16020180)
Supplement: Supplementary file 1 [file biomolecules-16-00180-s001.zip › biomolecules-4026598-supplementary.pdf]

Supplemental Materials:

LCMS-MS Measurement

To extract and measure T, E_2, P_4, CORT, androstenedione and estrone in hippocampal tissue, samples were homogenized in methanol containing internal standard using a sterile disposable pestle (DWK Life Sciences, Rockwood, TN). The samples were further homogenized using a MM400 ball mill (Retsch, Haan, Germany) operating at 30hz in three sessions of 5 minutes for a total of 15 minutes. They were homogenized a third time using a Polytron PT 10-35 homogenizer. The homogenate was cooled at -20°C for 30 minutes then centrifuged at 12,000 g for 5 minutes at 4°C. The resulting supernatant was evaporated to dryness and resuspended in 5% methanol for further purification. Steroid extraction and sample cleanup was achieved using an Oasis HLB solid phase extraction (Waters Corporation, Milford, MA). After cartridge conditioning and equilibration, the samples were loaded onto the cartridge and washed with 20% methanol. The steroids were eluted from the cartridge using 90% methanol. The final eluate was evaporated to dryness and derivatized with dansyl-chloride in acetone. The samples were then analyzed using a 6500+ triple quadrupole (Sciex, Toronto, Canada) adapted from the serum method. Intra and inter-assay CV was assessed with a pool of mouse hippocampi and ranged from 1.7 to 8.7 and 8.9 to 19.3%, respectively. Limit of detection (LOD) for T, E_2, P_4, and CORT for the hippocampi were 0.08, 0.004, 0.0036 and 0.7 pg/mg, respectively. For the plasma samples the LODs for T, E_2, P_4, and CORT were 0.003, 0.001, 0.003 and 0.2 pg/ml, respectively.
